# Rethinking gastric carcinogenesis: a multiscale ecological model of risk beyond *Helicobacter pylori*

**DOI:** 10.3389/fmicb.2026.1823306

**Published:** 2026-06-24

**Authors:** Juan Sebastián Frías-Ordoñez, José Darío Portillo-Miño, Hernando Marulanda-Fernandez, Yeison Carlosama, Lina Otero-Parra, José Augusto Urrego, Elder Otero-Ramos, William Otero Regino

**Affiliations:** 1 Department of Gastroenterology and Digestive Endoscopy, Faculty of Medicine, Universidad Nacional de Colombia, Bogotá, Colombia; 2 Gastroenterology and Digestive Endoscopy Unit, Gastroenterology Center, Bogotá, Colombia; 3 Hospital Internacional de Colombia, Bucaramanga, Colombia; 4 Colombian Research Group on Helicobacter pylori, Bogotá, Colombia; 5 Division of Research, Instituto Departamental de Salud de Nariño (IDSN), Pasto, Colombia; 6 Master’s Program in Epidemiology, Department of Public Health and Epidemiology, Faculty of Health Sciences, Pontificia Universidad Javeriana, Cali, Colombia; 7 Grupo de Investigación en Ciencias y Salud (GICIENSA), Faculty of Health Sciences, Medicine Program, Universidad de Nariño, Pasto, Colombia; 8 Hospital Universitario Departamental de Nariño, Pasto, Colombia; 9 Hospital Central de la Policía, Bogotá, Colombia; 10 Grupo Interdisciplinario de Investigación Salud-Enfermedad (GIISE), Universidad Cooperativa de Colombia, Pasto, Colombia

**Keywords:** cancer prevention, ecological framework, gastric cancer, global epidemiology, health disparities, *Helicobacter pylori*, microbiota

## Abstract

Gastric cancer remains one of the leading causes of cancer mortality worldwide, characterized by marked geographic disparities that cannot be fully explained by the distribution of *Helicobacter pylori* infection alone. Although *H. pylori* is recognized as the principal etiological agent, reductionist models centered exclusively on infection prevalence and eradication fail to account for the complex heterogeneity of gastric cancer risk across populations. In this narrative review, we propose a multiscale ecological framework that integrates infectious, host, environmental, microbial, and socioeconomic determinants to reinterpret gastric carcinogenesis as an emergent phenomenon arising from dynamic interactions across biological and geographic scales. Drawing on current epidemiological, molecular, and systems biology evidence, we examine how *H. pylori* interacts with host genetic susceptibility, epigenetic alterations, microbial community dynamics, dietary exposures, environmental modifiers, and structural social determinants to shape the trajectory of the Correa precancerous cascade. This integrative perspective helps explain key epidemiological paradoxes, including the persistence of high gastric cancer incidence in regions with comparable infection prevalence and the incomplete risk reduction observed after bacterial eradication in advanced mucosal injury. We further highlight the concept of persistent molecular and microenvironmental “carcinogenic memory,” whereby epigenetic alterations and dysbiotic microecological states sustain oncogenic potential even after elimination of the infectious agent. By framing gastric carcinogenesis as a complex ecological process rather than a pathogen-driven event, this model bridges mechanistic insights with population-level patterns and provides a conceptual platform for more effective prevention strategies. Ultimately, this framework supports a shift toward integrated approaches that combine early detection, targeted eradication, environmental modification, microbiome-aware strategies, and equitable health policies to reduce the global burden of gastric cancer.

## Introduction

Gastric cancer (GC) remains one of the leading causes of cancer-related mortality worldwide, ranking among the five most common and deadliest malignancies, with approximately 1.2–1.3 million new cases and nearly 950,000 deaths annually according to GLOBOCAN and Global Burden of Disease estimates (Sundar et al., 2025; Song et al., 2022; Zhou et al., 2025; Zhang et al., 2024). Despite declining age-standardized incidence and mortality rates over recent decades, the absolute global burden continues to rise due to population aging and growth, particularly in Asia (Song et al., 2022; Zhou et al., 2025; Zhang et al., 2024; Liu et al., 2025). GC also exhibits marked geographic heterogeneity, with the highest incidences observed in East Asia and substantially lower rates in highly developed regions, reflecting major disparities in risk-factor distribution, early detection programs, and healthcare-system capacity (Sundar et al., 2025; Zhou et al., 2025; Zhang et al., 2024; Liu et al., 2025; Mousavi et al., 2025).

*Helicobacter pylori* (*H. pylori*) infection is the principal etiological factor for GC, accounting for up to 90% of cases worldwide and an estimated attributable risk of 75–89% (Shah et al., 2025; Chey et al., 2024). Chronic infection initiates the Correa cascade, progressing from chronic gastritis and gastric atrophy to gastric intestinal metaplasia, dysplasia, and ultimately carcinoma, thereby providing a strong rationale for prevention strategies centered on early eradication (Chey et al., 2024). Randomized clinical trials and meta-analyses have shown that *H. pylori* eradication reduces GC incidence by approximately 46–50% and mortality by nearly 39%, particularly when treatment is administered before the development of premalignant gastric lesions (Shah et al., 2025; Chey et al., 2024; Liou et al., 2025; Argueta and Moss, 2021). These findings have supported population-based screening and eradication programs in high-incidence countries such as Japan and South Korea, where integration with endoscopic surveillance has improved early detection and contributed to declining mortality rates (Sundar et al., 2025; Chey et al., 2024; Chiang et al., 2025; Chiang et al., 2022). Recent international consensus statements further recommend adapting eradication and screening strategies to regional risk patterns and antibiotic resistance profiles, reinforcing the central role of *H. pylori* in GC prevention (Liou et al., 2025; Argueta and Moss, 2021; Chiang et al., 2025).

However, the persistence of GC despite widespread *H. pylori* eradication highlights important limitations of uniform prevention strategies. In many individuals, eradication occurs after the development of chronic atrophic gastritis or gastric intestinal metaplasia, lesions that maintain a substantial residual risk of progression even after elimination of the infection (Myeong et al., 2025). These premalignant changes are accompanied by persistent molecular alterations, including CpG island hypermethylation and aberrant microRNA expression, which are only partially reversible and support the need for risk-adapted endoscopic surveillance (Myeong et al., 2025). In addition, marked geographic heterogeneity in GC incidence among populations with similar *H. pylori* prevalence underscores the contribution of additional biological and environmental modifiers, including bacterial virulence factors such as cagA and vacA, host-pathogen coevolution, dietary patterns, and environmental exposures (De Sablet et al., 2011; Herrero et al., 2020; Namikawa et al., 2025; Kodaman et al., 2014). The variable effectiveness of eradication programs across regions—shaped by disease stage at intervention, antibiotic resistance, and healthcare inequalities—further suggests that uniform prevention strategies fail to capture the multilevel complexity of GC risk and progression (Myeong et al., 2025; Namikawa et al., 2025; Yadlapati and Shaheen, 2025).

Gastric cancer is a multifactorial disease in which infectious, environmental, dietary, genetic, microbial, and socioeconomic determinants interact dynamically along Correa’s precancerous cascade. Although *H. pylori* infection is the principal modifiable risk factor and initiator of progression from chronic atrophic gastritis to intestinal metaplasia, dysplasia, and carcinoma, only a minority of infected individuals ultimately develop GC, highlighting the contribution of additional biological and environmental modifiers Sundar et al., 2025. Dietary and lifestyle factors play a central role, as high consumption of salt, red and processed meats, and preserved foods is consistently associated with increased GC risk, whereas diets rich in fruits, vegetables, and fiber appear protective. Smoking and alcohol consumption further promote gastric inflammation and progression of premalignant lesions (Sundar et al., 2025; Shah et al., 2025). Genetic susceptibility also contributes to interindividual variability through inflammatory-response polymorphisms, hereditary cancer syndromes, and host-pathogen interactions, while alterations in the gastric and oral microbiota may amplify inflammatory and immune pathways involved in carcinogenesis, even in the absence of *H. pylori* (Sundar et al., 2025; Chiurillo, 2014; Huang et al., 2023). In addition, socioeconomic conditions influence both exposure to environmental risk factors and access to early diagnosis and treatment, contributing to persistent geographic and intergenerational disparities in GC burden (Shah et al., 2025; Sun et al., 2022).

Despite major advances in understanding gastric carcinogenesis, existing frameworks remain fragmented and often focus on isolated biological, environmental, or epidemiological domains. Although systems-based and multilevel models have provided important insights, integration of these determinants into a unified ecological framework linking molecular mechanisms, population heterogeneity, and geographically contextualized prevention strategies remains limited. Much of the current literature examines individual components of GC risk—such as *H. pylori* infection, molecular biomarkers, risk stratification models, or screening strategies—in relative isolation, without fully integrating biological, environmental, genetic, microbial, and socioeconomic determinants into a coherent multiscale framework for clinical and public health interpretation (Huang et al., 2023; Camargo et al., 2025). This fragmentation limits both comprehensive risk assessment and the ability to compare epidemiological patterns and prevention outcomes across populations with distinct ecological and healthcare contexts.

Consequently, GC prevention and screening strategies vary substantially across regions according to local epidemiology and healthcare-system capacity, limiting cross-population comparability and extrapolation of outcomes (Huang et al., 2023; Januszewicz et al., 2023). Although biomarker-based and multi-omics approaches are emerging as promising tools for individualized risk stratification, their large-scale implementation and validation across diverse populations remain limited (Yadlapati and Shaheen, 2025; Fan et al., 2024). International consensus statements have therefore emphasized the need for integrative, multidisciplinary approaches supported by large-scale data resources; however, available evidence remains largely regional and lacks a common conceptual platform for systematic comparison of risk and prevention strategies across geographic settings (Huang et al., 2023; Camargo et al., 2025; Januszewicz et al., 2023). In this context, the present review proposes a multiscale ecological framework for GC risk interpretation that integrates biological, environmental, microbial, and socioeconomic determinants into a unified conceptual structure. Rather than introducing a novel biological mechanism, this review aims to provide a critical integrative synthesis that connects existing evidence across molecular, environmental, and population-level domains to support more context-adapted and globally comparable prevention strategies.

## Literature search and evidence synthesis

### Study design

This narrative review adopted a conceptual and integrative approach to synthesize current evidence on gastric cancer (GC) epidemiology, risk factors, biological mechanisms, and prevention strategies, with the aim of developing a multiscale ecological framework for GC risk interpretation across individual, population, and geographic levels.

### Search strategy

A comprehensive literature search was conducted in PubMed/MEDLINE, Scopus, and Web of Science for studies published between January 2000 and January 2026, with selective inclusion of seminal earlier studies considered foundational to gastric carcinogenesis research. The search combined MeSH and free-text terms related to GC and its determinants, including “stomach neoplasms,” “gastric cancer,” “*Helicobacter pylori*,” “risk factors,” “precancerous lesions,” “epidemiology,” “screening,” “prevention,” “microbiota,” and “geographic variation.”

The retrieved literature underwent a two-stage screening process consisting of title/abstract review followed by full-text evaluation according to predefined thematic domains. Priority was given to high-quality evidence, including large cohort studies, randomized clinical trials, meta-analyses, translational mechanistic studies, international consensus statements, and authoritative reviews. Particular attention was paid to studies exploring mechanistic integration across biological scales, including interactions among microbial dysbiosis, inflammatory signaling, environmental exposures, epigenetic remodeling, and systems-level determinants of gastric carcinogenesis.

Although a formal risk-of-bias assessment was not performed due to the narrative and integrative nature of the review, evidence synthesis prioritized methodological robustness, consistency across studies, biological plausibility, translational relevance, and representation across diverse geographic, socioeconomic, and epidemiological contexts.

### Eligibility criteria

Included publications comprised original studies, systematic and narrative reviews, meta-analyses, clinical guidelines, and international consensus statements addressing one or more of the following domains: (i) GC epidemiology and global burden; (ii) biological mechanisms and progression along Correa’s cascade; (iii) infectious, environmental, dietary, genetic, microbial, and socioeconomic risk factors; (iv) *H. pylori* prevention, screening, and eradication strategies; and (v) regional or population differences in GC risk and outcomes.

Case reports, non-peer-reviewed publications, and studies lacking relevance to GC risk stratification, prevention, or population-level interpretation were excluded.

### Selection of studies and evidence synthesis

Titles and abstracts were screened for relevance to the review objectives, followed by full-text assessment of selected articles. Given the conceptual and narrative nature of the review, no formal methodological quality scoring system was applied. Instead, evidence synthesis emphasized high-quality studies, large population-based analyses, randomized trials, meta-analyses, and international guidelines. Findings were synthesized qualitatively, focusing on consistency of evidence, biological plausibility, mechanistic integration, and relevance across diverse epidemiological and geographic settings.

### Development of the conceptual framework

Based on the integrated synthesis of selected literature, a complementary ecological framework was developed to interpret GC risk across interconnected biological, environmental, socioeconomic, and healthcare-system scales. The framework was informed by established models of gastric carcinogenesis, including Correa’s cascade, and iteratively refined to highlight dynamic interactions among microbial, inflammatory, environmental, and contextual determinants relevant to prevention and risk stratification strategies.

### Ethical considerations

As this study was based exclusively on previously published literature, ethics committee approval and informed consent were not required.

## Results

### Geographic gradients of gastric cancer risk

GC exhibits marked geographic gradients that persist even after adjustment for age and sex. The highest incidence rates are concentrated in East Asia—particularly Japan, South Korea, and China—as well as in Andean regions of South America and Eastern Europe, whereas North America, sub-Saharan Africa, and Oceania show substantially lower burdens (Sundar et al., 2025; Wong et al., 1980; Morgan et al., 2022; López et al., 2023). The temporal and geographic stability of these patterns suggests that GC risk is not randomly distributed, but rather emerges from region-specific ecological configurations shaped by interacting biological, environmental, and contextual determinants (Sundar et al., 2025; Wong et al., 1980; Morgan et al., 2022; López et al., 2023).

**Figure 1:**
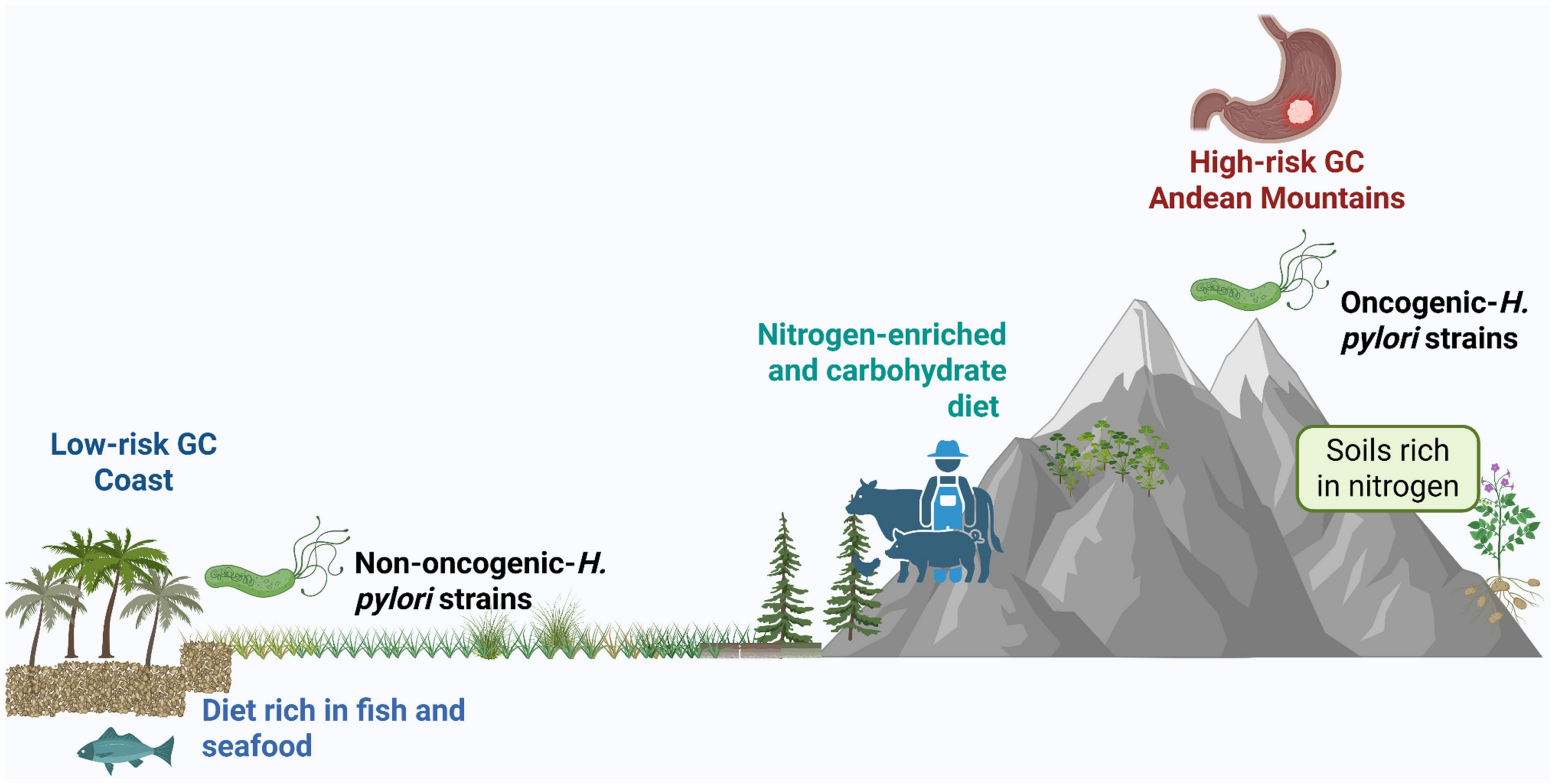
Eco-epidemiological framework linking geographic, environmental, dietary, and microbial factors to differential gastric cancer (GC) risk. Coastal regions characterized by fish-rich diets and predominance of non-oncogenic *H. pylori* strains are associated with lower GC incidence. In contrast, high-altitude Andean regions with nitrogen-enriched soils, carbohydrate-dominant diets, and circulation of oncogenic *H. pylori* strains exhibit increased GC risk. This model illustrates how geographic and environmental contexts may modulate microbial virulence patterns and dietary exposures, contributing to regional disparities in gastric carcinogenesis. Source: created with www.bioRender.com.

This ecological organization of risk becomes particularly evident when comparing regions with similar *Helicobacter pylori* prevalence but markedly different GC burdens (Figure 1) (Wong et al., 1980; Morgan et al., 2022; Wang et al., 2015). The resulting “ecological discrepancy” between infection and cancer—classically illustrated by the “African enigma” and observations from South America and Asia—indicates that *H. pylori* infection alone is insufficient to explain population-level risk (Wong et al., 1980; Morgan et al., 2022; Wang et al., 2015). Intra-regional evidence from countries such as Chile further demonstrates that populations with comparable infection rates and bacterial virulence profiles may still exhibit contrasting GC incidences associated with differences in gastric atrophy prevalence and dietary exposures (Herrero et al., 2020). Collectively, these findings support the need for integrative frameworks capable of interpreting GC risk as the product of dynamic interactions among microbial, host, environmental, and contextual factors rather than isolated determinants (Wong et al., 1980; Morgan et al., 2022; Wang et al., 2015).

Examples of geographic heterogeneity in GC burden and their proposed ecological determinants are summarized in Table 1.

**Table 1:** Examples of geographic heterogeneity in gastric cancer risk and proposed ecological determinants.

Region	*H. pylori* prevalence	GC burden	Proposed contributing factors
East Asia	High	Very high	Virulent strains, salt-rich diets, aging
Sub-Saharan Africa	High	Low	African enigma, microbiome/environment
Andes region	High	High	Altitude, diet, socioeconomic factors

Geographic patterns shown are based on epidemiological observations reported in high-incidence and low-incidence regions worldwide. Proposed ecological determinants represent integrative interpretations derived from the reviewed literature and should not be interpreted as isolated causal relationships. Source: Prepared by the authors based on epidemiological and ecological evidence discussed in references Sundar et al. (2025), Song et al. (2022), Zhou et al. (2025), Zhang et al. (2024), Liu et al. (2025), Mousavi et al. (2025), Herrero et al. (2020), Wong et al. (1980), Morgan et al. (2022), López et al. (2023), Wang et al. (2015), Park et al. (2019), Yin et al. (2020), Beach et al. (2026), Correa et al. (1975), Correa (1992), Mirvish (1995), Kobayashi (2018), Palli et al. (2001), Donat-Vargas et al. (2025), and Kayamba and Kelly (2022).

### Biological and infectious determinants at the individual level

**Figure 2:**
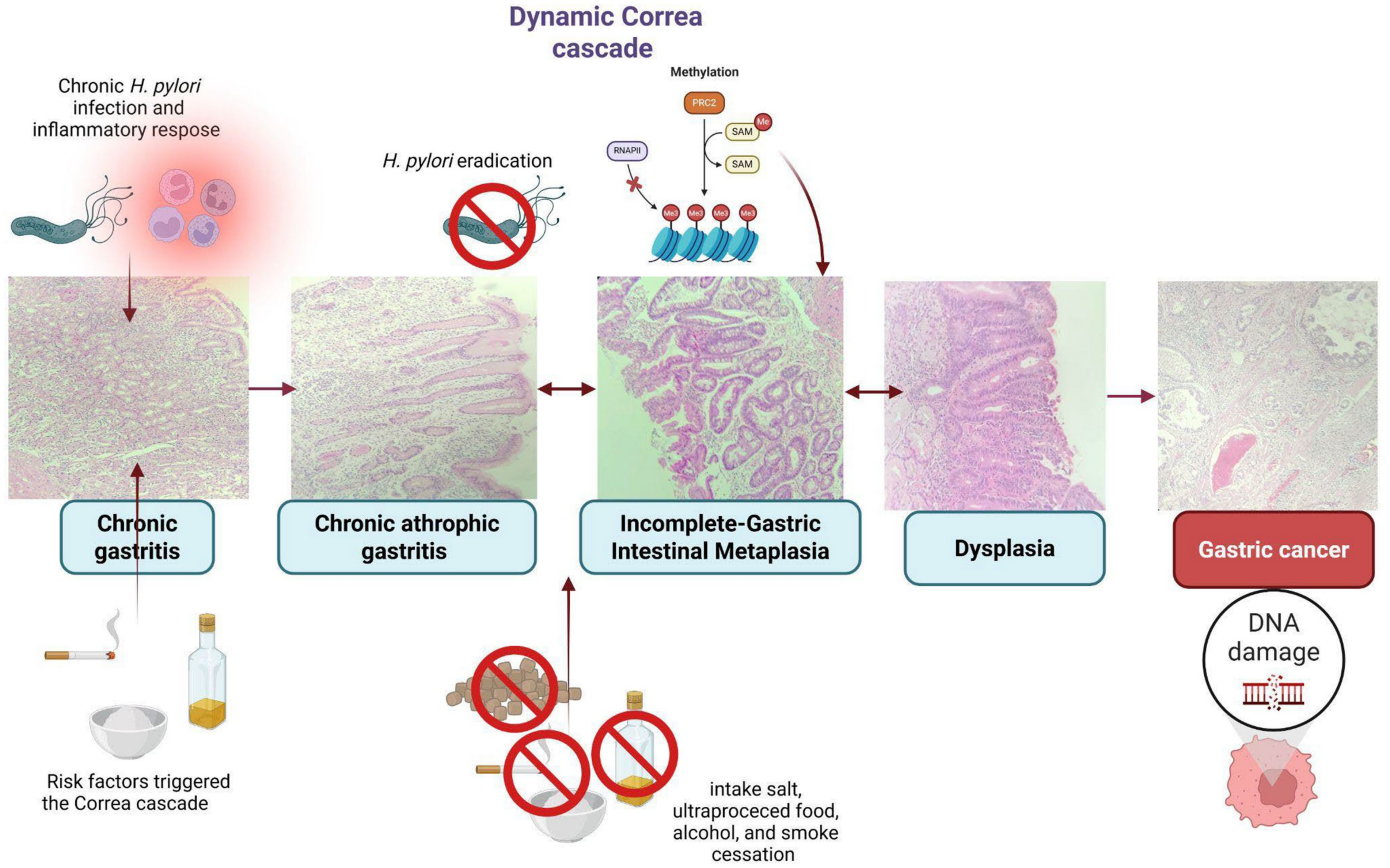
Multistep gastric carcinogenesis according to the dynamic Correa cascade and its environmental and microbial modulators. Chronic *H. pylori* infection initiates non-atrophic gastritis, which may progress to chronic atrophic gastritis, intestinal metaplasia, dysplasia, and ultimately gastric cancer. Early eradication of *H. pylori* may interrupt or partially reverse precancerous stages; however, progression becomes less reversible after the establishment of intestinal metaplasia. Environmental exposures, including high salt intake, ultra-processed diet, smoking, and alcohol consumption, act as modulators that accelerate mucosal damage and genomic instability. Accumulation of DNA damage and epigenetic alterations contributes to malignant transformation. Bidirectional arrows indicate dynamic interactions and potential transitional plasticity within early stages of the cascade. Source: created with www.bioRender.com.

Gastric carcinogenesis follows the sequential progression described in Correa’s cascade (Figure 2), evolving from chronic non-atrophic gastritis to chronic atrophic gastritis, intestinal metaplasia, dysplasia, and ultimately adenocarcinoma (Sundar et al., 2025; Chey et al., 2024). *H. pylori* infection acts as the principal initiating factor by inducing persistent inflammation and promoting molecular and epigenetic alterations that drive histological progression (Sundar et al., 2025; Chey et al., 2024; Zhou et al., 2025). Early stages of the cascade, particularly non-atrophic gastritis and incipient atrophy, may be partially reversible following *H. pylori* eradication, especially when intervention occurs before advanced mucosal remodeling develops (Zhou et al., 2025; Cheng et al., 2023; Xi et al., 2023). However, once advanced premalignant lesions such as intestinal metaplasia or dysplasia are established, reversibility becomes limited and GC risk persists despite bacterial eradication, reflecting cumulative biological damage over time (Myeong et al., 2025; Cheng et al., 2023).

Longitudinal studies further show that late *H. pylori* eradication reduces—but does not eliminate—GC risk because stable genetic and epigenetic alterations may persist after progression through intermediate and advanced stages of Correa’s cascade (Sundar et al., 2025; Myeong et al., 2025; Cheng et al., 2023). These alterations include CpG island hypermethylation and aberrant microRNA expression, particularly in areas of intestinal metaplasia, which maintain a mucosal phenotype susceptible to malignant transformation even after clearance of active infection (Sundar et al., 2025; Myeong et al., 2025; Valenzuela et al., 2015). This persistent molecular imprint has been described as a “mucosal memory of damage,” whereby prior inflammatory injury sustains long-term oncogenic susceptibility (Myeong et al., 2025; Valenzuela et al., 2015). Consequently, although early *H. pylori* eradication remains a cornerstone of primary prevention, patients with advanced premalignant lesions often require prolonged endoscopic surveillance and risk stratification based on the extent of atrophy and intestinal metaplasia, underscoring that effective GC prevention extends beyond bacterial eradication alone (Myeong et al., 2025; Cheng et al., 2023).

### Environmental, dietary, and microbiological modulators

Diet and lifestyle behaviors are major modulators of gastric carcinogenesis, exerting both independent and synergistic effects with *H. pylori* infection along Correa’s cascade. High consumption of salt, processed meats, and traditionally preserved foods is consistently associated with increased GC risk (Sundar et al., 2025; Shah et al., 2025; Maddineni et al., 2022; Raei et al., 2016). Salt contributes directly to mucosal injury by disrupting the gastric barrier, enhancing *H. pylori* virulence, and facilitating penetration of carcinogenic compounds, thereby amplifying chronic inflammation and oxidative stress (Sundar et al., 2025; Shah et al., 2025; Maddineni et al., 2022; Raei et al., 2016). Processed and preserved foods further promote the formation of N-nitroso compounds, whose carcinogenic effects are intensified within chronically inflamed gastric tissue (Sundar et al., 2025; Maddineni et al., 2022; Raei et al., 2016). In contrast, diets rich in fruits, vegetables, and fiber appear protective by providing antioxidants and phytochemicals that reduce oxidative damage and modulate inflammatory signaling (Shah et al., 2025; Maddineni et al., 2022; Raei et al., 2016). Smoking and alcohol consumption act as additional cofactors, synergistically increasing GC risk in the presence of *H. pylori* infection (Sundar et al., 2025; Collatuzzo et al., 2021).

Importantly, these interactions operate across interconnected biological scales rather than as isolated processes. Environmental and dietary exposures may reshape gastric ecological networks by altering microbial composition, metabolic activity, and mucosal inflammatory tone, thereby promoting oxidative stress, carcinogenic metabolite production, and epigenetic instability. These cumulative alterations progressively reinforce a pro-carcinogenic gastric microenvironment.

Beyond classic dietary exposures, increasing evidence identifies the gastric microbiota as a dynamic ecological network involved in carcinogenesis beyond *H. pylori* alone. The healthy stomach harbors a diverse microbial ecosystem dominated by Firmicutes, Proteobacteria, Actinobacteria, Bacteroidetes, and Fusobacteria, which undergoes substantial ecological disruption along Correa’s cascade (Zeng et al., 2024; Bautista et al., 2025; Wizenty and Sigal, 2025). Progression from chronic gastritis to atrophic gastritis, intestinal metaplasia, dysplasia, and GC is accompanied by loss of gastric acidity and progressive dysbiosis, facilitating colonization by oral-derived pathobionts and non-*H. pylori* microorganisms associated with increased carcinogenic risk (Bessède and Mégraud, 2022; Rajilic-Stojanovic et al., 2020). Meta-analyses further demonstrate reduced microbial *α*-diversity and compositional shifts characterized by enrichment of Fusobacterium, Parvimonas, Veillonella, Prevotella, Peptostreptococcus, and Lactobacillus, alongside depletion of commensal genera such as Bifidobacterium and Blautia (Yang et al., 2021; Liu and Shah, 2022; Coker et al., 2018).

Recent evidence suggests that carcinogenic potential depends not only on the presence of specific microbial taxa, but also on ecological network reorganization and metabolic remodeling within the gastric microenvironment. Co-occurrence network analyses demonstrate strengthened interactions among GC-enriched bacteria and disruption of commensal microbial networks during disease progression, supporting the concept of a gastric “oncobiome” characterized by coordinated pro-inflammatory and pro-tumoral interactions (Coker et al., 2018; Park et al., 2019). Multi-omics studies integrating microbiome and metabolomic profiling further identify gastric atrophy as a critical metabolic transition marked by depletion of anti-inflammatory short-chain fatty acids and accumulation of pro-inflammatory and nitrosating metabolites (Park et al., 2019; Yang et al., 2026).

The oral-gastric microbial axis has also emerged as an important contributor to gastric carcinogenesis. Oral pathobionts such as *Porphyromonas gingivalis* and *Fusobacterium nucleatum* may colonize the stomach under altered gastric conditions through salivary, hematogenous, or mucosal routes (He et al., 2026; Wu et al., 2026; Zha et al., 2026). Once established, these organisms amplify inflammation through activation of NF-κB and Wnt/*β*-catenin pathways, promote immune dysregulation via IL-1β, IL-6, and IL-17 signaling, generate genotoxic metabolites, and contribute to biofilm formation and epithelial dysfunction (He et al., 2026; Zha et al., 2026). Observational studies further suggest that a proportion of chronic atrophic gastritis cases may occur independently of *H. pylori* infection, supporting the concept that oral-derived dysbiosis may contribute to carcinogenic progression beyond classical infection-centered models (Zha et al., 2026; Song et al., 2026).

Experimental and translational studies further support mechanistic links between dysbiosis and gastric carcinogenesis. Germ-free and gnotobiotic murine models demonstrate that microbial communities beyond *H. pylori* can independently modulate tumor-promoting inflammation, immune remodeling, oxidative stress, and metabolic reprogramming (Wizenty and Sigal, 2025; Bessède and Mégraud, 2022). Dysbiosis-associated carcinogenesis has been linked to chronic activation of TLR/NLR and NF-κB pathways, increased production of N-nitroso compounds, depletion of protective metabolites, expansion of immunosuppressive cell populations, and epigenetic remodeling driven by oxidative DNA damage and inflammatory signaling (Bautista et al., 2025; Mohamed et al., 2025; Kaźmierczak-Siedlecka et al., 2022; Wu et al., 2024; Gao et al., 2026; Liu et al., 2022).

Nevertheless, although accumulating evidence supports a role for gastric dysbiosis in carcinogenesis, most available studies remain observational and cross-sectional, limiting causal inference. It remains uncertain whether many reported microbial alterations represent true carcinogenic drivers or secondary ecological shifts resulting from hypochlorhydria and tumor-associated microenvironmental remodeling.

The principal multiscale determinants and mechanistic pathways implicated in gastric carcinogenesis are summarized in Table 2.

**Table 2:** Multiscale determinants and mechanistic pathways involved in gastric carcinogenesis.

Scale/domain	Major determinants	Proposed mechanisms	Translational implications
Microbial	*H. pylori*, dysbiosis, oral pathobionts	Chronic inflammation, NF-κB activation, nitrosation	Eradication, microbiome-targeted prevention
Genetic	IL-1, TNF polymorphisms, CDH1	Immune dysregulation, susceptibility	Risk stratification
Epigenetic	CpG methylation, miRNAs	Epigenetic memory, field defect	Biomarker development
Environmental	Salt, PAHs, smoking	Oxidative stress, mutagenesis	Exposure reduction
Socioeconomic	Poverty, healthcare access	Delayed diagnosis, chronic exposure	Screening prioritization

This table summarizes the principal biological, environmental, microbial, epigenetic, socioeconomic, and healthcare-system determinants implicated in gastric carcinogenesis according to the integrative ecological framework proposed in this review. Mechanistic pathways and translational implications were synthesized from epidemiological, mechanistic, translational, and systems-level studies discussed throughout the manuscript. Source: Prepared by the authors based on evidence synthesized from references Sundar et al. (2025), Shah et al. (2025), Myeong et al. (2025), Morgan et al. (2022), Zeng et al. (2024), Bautista et al. (2025), Wizenty and Sigal (2025), Bessède and Mégraud (2022), Rajilic-Stojanovic et al. (2020), Yang et al. (2021), Liu and Shah (2022), Coker et al. (2018), Park et al. (2019), Yang et al. (2026), El-Omar et al. (2000), Machado et al. (2003), Persson et al. (2011), Zeyaullah et al. (2021), Negovan et al. (2019), Ushijima and Hattori (2012), Maeda et al. (2017), Niwa et al. (2010), Baba et al. (2016), Kiga et al. (2014), Ando et al. (2009), Takeshima et al. (2015), Yoshida et al. (2017), Woo et al. (2018), Michigami et al. (2018), Watari et al. (2019), Tanaka et al. (2022), Sudo et al. (2025), Yamada et al. (2025), Chang et al. (2026), Vineis et al. (2020), DeBord et al. (2016), Bray et al. (2024), Correa et al., 1975, Correa (1992), Mirvish (1995), Kobayashi (2018), Palli et al. (2001), Donat-Vargas et al. (2025), Kayamba and Kelly (2022), Jayakrishnan and Ng (2025), Liang et al. (2025), Hui et al. (2023), Li et al. (2026), Sarigiannis et al. (2026), Juarez and Matthews-Juarez (2018), Usui et al. (2023), and Jin et al. (2020).

### Genetic and epigenetic factors

Emerging evidence suggests that microbial dysbiosis contributes to gastric carcinogenesis not only through chronic inflammation, but also through epigenetic remodeling mediated by oxidative stress, microbial metabolites, immune signaling, and activation of DNA methyltransferase pathways. Host genetic susceptibility further modulates GC risk through polymorphisms in inflammatory-response genes. Variants in the IL-1 cluster, including IL-1B-511 and IL-1RN polymorphisms, are associated with increased IL-1β production, enhanced inflammation, reduced gastric acid secretion, and greater progression to chronic atrophic gastritis and GC in individuals with *H. pylori* infection (El-Omar et al., 2000; Machado et al., 2003; Persson et al., 2011). Similarly, the TNF-*α*-308A allele and combinations of multiple pro-inflammatory polymorphisms appear to increase risk additively, supporting a context-dependent polygenic susceptibility model (Machado et al., 2003; Zeyaullah et al., 2021). Other cytokine-related genes, including IL-10, IL-8, IL-18RAP, and IL-22, have also been associated with premalignant gastric lesions, although findings remain heterogeneous across populations (Persson et al., 2011; Negovan et al., 2019).

Importantly, many candidate-gene associations have shown inconsistent reproducibility across ethnic and geographic settings, suggesting that the contribution of host genetics is strongly modulated by environmental and microbial interactions. In contrast, hereditary syndromes such as CDH1-associated hereditary diffuse gastric cancer account for only a small proportion of total GC burden, reinforcing the concept that host genetics primarily modifies susceptibility within a multifactorial carcinogenic process rather than determining disease independently (Sundar et al., 2025).

Epigenetics represents a critical mechanistic bridge linking chronic inflammation, environmental exposures, microbial dysbiosis, and cumulative carcinogenic risk. Increasing evidence indicates that gastric carcinogenesis involves progressive inflammation-induced epigenetic reprogramming, generating an “epigenetic field defect” characterized by widespread aberrant DNA methylation in histologically non-neoplastic mucosa (Ushijima and Hattori, 2012; Maeda et al., 2017; Niwa et al., 2010). *H. pylori* infection, together with dietary and environmental exposures such as smoking, alcohol consumption, and high-salt intake, promotes cumulative CpG island hypermethylation in tumor suppressor genes including CDH1, RUNX3, FAT4, and p16/CDKN2A, as well as methylation-mediated silencing of microRNAs such as miR-124a, miR-34c, and miR-137 (Ushijima and Hattori, 2012; Baba et al., 2016; Kiga et al., 2014; Ando et al., 2009; Takeshima et al., 2015; Yoshida et al., 2017). Genome-wide analyses further suggest that carcinogenesis-related pathways are altered more frequently through aberrant methylation than somatic mutation, supporting epigenetic remodeling as an early and central driver of gastric carcinogenesis (Maeda et al., 2017).

These alterations progressively accumulate along Correa’s cascade and may persist long after *H. pylori* eradication, generating a phenomenon termed “epigenetic memory” (Niwa et al., 2010; Woo et al., 2018; Michigami et al., 2018; Watari et al., 2019). Experimental studies demonstrate that *H. pylori*-induced methylation includes both reversible and irreversible components, with stable methylation patterns persisting despite bacterial clearance (Niwa et al., 2010). Clinically, this persistence is particularly evident in intestinal metaplasia, where epigenetic alterations remain biologically fixed and continue to confer residual cancer risk after eradication therapy (Michigami et al., 2018; Watari et al., 2019; Tanaka et al., 2022). This dysregulated epigenetic landscape includes both CpG island hypermethylation and coordinated non-CpG hypomethylation near oncogenic loci such as CD44, CDH17, and HNF4A, supporting the existence of a persistent field defect that maintains long-term tissue vulnerability (Sudo et al., 2025). Longitudinal studies further demonstrate that methylation burden in noncancerous mucosa may stratify future GC risk following eradication therapy, highlighting the emerging translational potential of epigenetic biomarkers (Yamada et al., 2025).

Nevertheless, despite promising findings, the clinical implementation of epigenetic biomarkers remains limited by lack of methodological standardization, inter-platform variability, and insufficient prospective validation across diverse populations. Mechanistically, chronic inflammation itself appears to be the principal driver of epigenetic remodeling rather than *H. pylori* directly (Maeda et al., 2017; Niwa et al., 2010; Chiba et al., 2012). Persistent activation of NF-κB, IL-6/JAK/STAT, IL-8, and TGF-*β* signaling pathways promotes aberrant DNA methyltransferase activity, oxidative DNA damage, chromatin remodeling, genomic instability, and activation-induced cytidine deaminase–mediated mutagenesis (Matsumoto et al., 2007; Shimizu et al., 2012; Matsumoto et al., 2010; Nagata et al., 2014; Luan et al., 2025; Li et al., 2025; Liu et al., 2023; Nagaraju et al., 2026). Recent evidence also highlights the importance of metabolic-epigenetic interactions, particularly histone lactylation associated with lactate accumulation during the Warburg effect. H3K18 lactylation has been linked to oncogenic signaling, PD-L1-mediated immune evasion, suppression of CD8 + T-cell cytotoxicity, and amplification of tumor-promoting inflammatory pathways in GC (Yang et al., 2026; Chen et al., 2026; Qian et al., 2026).

### Socioeconomic and health system determinants

Socioeconomic factors constitute a fundamental structural determinant of GC risk by modulating both exposure to carcinogenic factors and disease progression. Poverty, overcrowding, limited access to healthy food, and inadequate healthcare infrastructure are consistently associated with higher GC incidence, reflecting the convergence of high-salt and ultra-processed diets, increased *H. pylori* prevalence, smoking, and obesity in socially vulnerable populations (Morgan et al., 2022). Overcrowding and adverse living conditions further facilitate early and intergenerational transmission of *H. pylori* infection, sustaining chronic inflammation and prolonged exposure to carcinogenic environments (Morgan et al., 2022). These cumulative exposures introduce a temporal and social dimension to GC risk, whereby structural disadvantage becomes biologically embedded through chronic exposure to inflammatory diets, microbial dysbiosis, environmental pollutants, psychosocial stressors, and limited healthcare access. Over time, this accumulated burden may interact with host susceptibility to promote epigenetic instability, immune dysfunction, and progression along Correa’s cascade.

Healthcare-system performance further determines how biological risk translates into clinical outcomes. Countries with organized endoscopic screening programs, such as Japan and South Korea, have achieved substantial increases in early-stage GC detection and five-year survival rates exceeding 70%, compared with rates below 35% in regions lacking structured early detection strategies (Shah et al., 2025; Morgan et al., 2022; Sun et al., 2024; Patel et al., 2026). In South Korea, endoscopic screening has been associated with significant reductions in GC mortality, while Japan has demonstrated consistent survival benefits in screened populations (Morgan et al., 2022; Sun et al., 2024; Patel et al., 2026). These observations illustrate how similar biological risk profiles—including high *H. pylori* prevalence and genetic susceptibility—may produce markedly different outcomes depending on healthcare access, screening infrastructure, and public health organization. Collectively, these findings reinforce that effective GC prevention depends not only on biomedical interventions, but also on policies addressing healthcare equity, access, and system-level capacity.

### Multiscale integration: the ecological framework of risk

**Figure 3:**
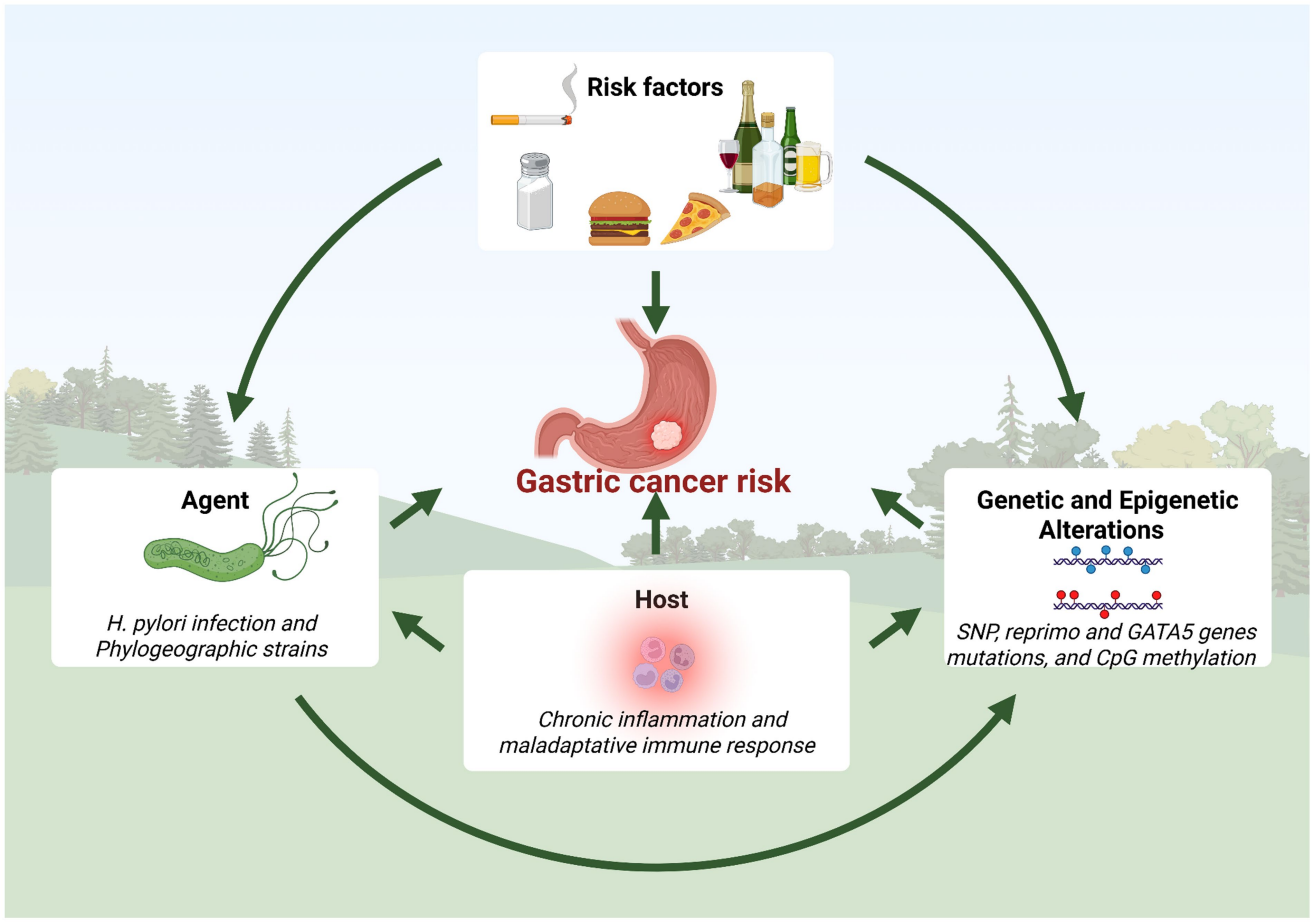
Integrated multilevel model of environmental, infectious, host inflammatory, and genetic/epigenetic determinants contributing to gastric carcinogenesis. Environmental exposures (smoking, alcohol consumption, ultra-processed diet) together with *H. pylori* infection trigger persistent gastric inflammation and immune dysregulation, leading to oxidative stress, genomic instability, and cumulative DNA damage. These processes promote the progressive accumulation of genetic (e.g., SNPs, tumor suppressor gene mutations) and epigenetic alterations (e.g., CpG island methylation, GATA5 silencing), ultimately increasing gastric cancer risk. Solid arrows indicate established causal relationships; bidirectional arrows represent feedback interactions within the tumor-promoting microenvironment. Source: created with www.bioRender.com.

GC risk should be understood as a complex emergent phenomenon arising from dynamic interactions across multiple biological and social levels rather than as the linear sum of independent determinants (Figure 3). At the individual level, susceptibility is influenced by host genetics, *H. pylori* infection, Epstein–Barr virus, immune status, smoking, alcohol consumption, and high-salt diets (Sundar et al., 2025; Morgan et al., 2022; Patel et al., 2026). However, progression to clinical disease depends critically on the gastric microenvironment, where chronic inflammation, microbial dysbiosis, environmental pollutants, hypoxia, and oxidative stress interact to promote tissue remodeling and carcinogenic progression (Sundar et al., 2025; Reytor-González et al., 2025). These processes occur within broader population and geographic contexts shaped by social determinants, living conditions, bacterial virulence patterns, and environmental exposures, generating marked spatial and temporal heterogeneity in GC incidence and mortality (Shah et al., 2025; Morgan et al., 2022; Yin et al., 2020; Beach et al., 2026). Within this framework, risk emerges through nonlinear and synergistic interactions that accumulate over time and across generations, progressively reshaping the gastric microenvironment toward carcinogenesis.

Chronic inflammation may function as a central systems-level integrative hub through which infectious, microbial, environmental, and metabolic exposures converge. Persistent inflammatory signaling not only sustains epithelial injury, but also alters immune responses, disrupts microbial ecological stability, promotes oxidative DNA damage, and facilitates epigenetic and metabolic reprogramming. These bidirectional interactions may progressively stabilize carcinogenic states within the gastric microenvironment, even after elimination of the initiating infectious trigger.

From a systems epidemiology perspective, gastric carcinogenesis is increasingly interpreted through an exposome-informed model in which infectious, dietary, chemical, microbial, environmental, socioeconomic, and host-related exposures interact across the life course (Sundar et al., 2025; Chang et al., 2026; Vineis et al., 2020). Rather than resulting from a single etiologic factor, GC emerges from the cumulative convergence of multiple external and internal exposures operating across temporal, biological, and geographic scales. Within this framework, the exposome encompasses three interconnected domains: the general external domain, including socioeconomic conditions, urbanization, climate, and geography; the specific external domain, including diet, smoking, alcohol consumption, infections, occupational exposures, medications, and pollutants; and the internal domain, integrating inflammation, oxidative stress, microbiome dynamics, metabolic alterations, and epigenetic remodeling (Vineis et al., 2020; DeBord et al., 2016). This perspective helps explain why only a minority of *H. pylori*-infected individuals ultimately develop GC, emphasizing that carcinogenesis depends on interactions among microbial virulence, host susceptibility, environmental exposures, and contextual determinants rather than infection alone (Sundar et al., 2025; Patel et al., 2026; Bray et al., 2024).

Environmental carcinogenesis in GC involves multiple convergent mechanisms including N-nitroso compounds, polycyclic aromatic hydrocarbons, high-salt exposure, chronic inflammation, and microbiome-mediated metabolic dysregulation. Chronic exposure to N-nitroso compounds within the hypochlorhydric stomach contributes to DNA alkylation and mutagenesis, a process amplified by nitrate-reducing bacteria and modulated by dietary antioxidants such as vitamin C and polyphenols (Correa et al., 1975; Correa, 1992; Mirvish, 1995; Kobayashi, 2018; Palli et al., 2001). Integrative multi-omics studies further identify PAH-associated carcinogenic signatures linked to immune suppression, invasive phenotypes, and poor survival, suggesting substantial geographic variability in environmental carcinogenesis according to local exposure ecologies (Park et al., 2019). Additional evidence implicates biomass smoke, particulate matter, waterborne nitrate exposure, occupational hazards, and adverse built environments as contributors to GC risk (Shah et al., 2025; Wong et al., 1980; Donat-Vargas et al., 2025; Kayamba and Kelly, 2022). Several observational and Mendelian randomization studies further suggest that the effects of high salt intake, smoking, obesity, alcohol consumption, and dietary patterns are mediated through inflammatory, microbial, and metabolic pathways rather than isolated linear associations (Patel et al., 2026; Jayakrishnan and Ng, 2025; Liang et al., 2025; Hui et al., 2023; Li et al., 2026).

Nevertheless, the consistency of associations between environmental exposures and GC varies substantially across populations, reflecting methodological heterogeneity, differences in exposure assessment, and complex interactions with genetic and microbial factors. Systems epidemiology and multi-omics integration now provide increasingly sophisticated tools for understanding these interactions across scales. Recent studies integrating genomics, microbiomics, metabolomics, proteomics, epigenomics, and exposure profiling suggest that GC comprises biologically heterogeneous exposure-informed subtypes with distinct molecular and immunologic characteristics (Chang et al., 2026; Sarigiannis et al., 2026; Juarez and Matthews-Juarez, 2018). Gene–environment interaction studies further demonstrate that inherited susceptibility may amplify the carcinogenic effects of environmental exposures (Usui et al., 2023), whereas large polygenic-risk analyses suggest that healthy lifestyle patterns can attenuate GC risk even among genetically susceptible individuals (Jin et al., 2020).

This multiscale ecological approach provides both a conceptual and operational framework for interpreting divergent GC outcomes across populations with apparently similar risk profiles. By integrating individual, microenvironmental, population-level, and geographic determinants, the model may help prioritize prevention strategies according to local context, including targeted *H. pylori* eradication, environmental risk reduction, and screening programs adapted to population risk (Shah et al., 2025; Morgan et al., 2022; Yin et al., 2020; Beach et al., 2026). In this perspective, GC prevention should be understood not as a uniform intervention, but as a dynamic and context-specific process aligned with the ecological characteristics of each population. Nevertheless, although the proposed framework integrates evidence across multiple biological and population domains, several relationships remain associative and context-dependent rather than definitively causal. Accordingly, the model should be interpreted as an integrative and hypothesis-generating framework intended to support future translational and epidemiological research rather than as a deterministic representation of gastric carcinogenesis. Potential prevention and surveillance strategies according to different ecological risk configurations are summarized in Table 3.

**Table 3:** Potential prevention strategies according to ecological and biological risk profiles.

Risk configuration	Dominant determinants	Suggested strategies
High *H. pylori* prevalence	Infection transmission	Population eradication
Advanced GIM/CAG	Epigenetic persistence	Endoscopic surveillance
High environmental exposure	Salt, smoking, pollutants	Public health interventions
Low-resource settings	Healthcare limitations	Risk-prioritized screening

Proposed prevention strategies are conceptual and intended to illustrate how the ecological framework may support context-adapted risk stratification and prevention planning across diverse biological and healthcare-system settings. Recommendations are derived from evidence discussed throughout the review and are not intended to replace formal clinical guidelines. Source: Prepared by the authors based on evidence and prevention strategies discussed in references Shah et al. (2025), Chey et al. (2024), Liou et al. (2025), Argueta and Moss (2021), Chiang et al. (2025), Chiang et al. (2022), Myeong et al. (2025), Yadlapati and Shaheen (2025), Huang et al. (2023), Januszewicz et al. (2023), Fan et al. (2024), Cheng et al. (2023), Michigami et al. (2018), Watari et al. (2019), Tanaka et al. (2022), Sudo et al. (2025), Yamada et al. (2025), Sun et al. (2024), Patel et al. (2026), Usui et al. (2023), and Jin et al. (2020).

## Conclusion

Gastric cancer should be understood as a complex multiscale disease arising from dynamic interactions among microbial, genetic, environmental, metabolic, and socioeconomic determinants rather than from isolated etiological factors alone. The available evidence indicates that gastric carcinogenesis is shaped by cumulative and nonlinear processes operating across biological and population levels, in which chronic inflammation functions as a central integrative mechanism linking *H. pylori* infection, microbial dysbiosis, environmental exposures, immune remodeling, and epigenetic instability along Correa’s cascade.

Although *H. pylori* eradication remains a cornerstone of primary prevention, its effectiveness is strongly conditioned by the stage of mucosal damage at intervention, as persistent epigenetic and microenvironmental alterations may sustain residual oncogenic risk despite bacterial clearance. These observations help explain the marked geographic heterogeneity of GC incidence and the discordance between *H. pylori* prevalence and cancer burden observed across populations.

In this context, the proposed ecological framework provides an integrative and operational structure for interpreting GC risk across interconnected biological, environmental, and healthcare-system scales. By linking mechanistic pathways with population-level and geographic determinants, the model may support risk-adapted prevention strategies, including targeted eradication programs, context-specific surveillance, environmental risk reduction, and precision prevention approaches tailored to regional epidemiological realities.

Nevertheless, several interactions within the proposed framework remain associative and incompletely characterized, highlighting the need for further translational, longitudinal, and multi-omics research to clarify cross-scale causal mechanisms and validate scalable biomarkers for individualized risk stratification and early detection. Ultimately, sustainable reduction of the global GC burden will require not only biomedical interventions, but also coordinated public health policies addressing healthcare inequities, environmental exposures, and access to early diagnosis and prevention.
